# Past innovations and future possibilities in plant chromosome engineering

**DOI:** 10.1111/pbi.14530

**Published:** 2024-11-29

**Authors:** Yang Liu, Qian Liu, Congyang Yi, Chang Liu, Qinghua Shi, Mian Wang, Fangpu Han

**Affiliations:** ^1^ Institute of Genetics and Developmental Biology Chinese Academy of Sciences Beijing China; ^2^ University of the Chinese Academy of Sciences Beijing China; ^3^ Center for Plant Biology, School of Life Sciences Tsinghua University Beijing China; ^4^ Tsinghua University‐Peking University Joint Center for Life Sciences, School of Life Sciences Tsinghua University Beijing China

**Keywords:** Plant chromosome engineering, centromere, minichromosome, CRISPR/Cas technology

## Abstract

Plant chromosome engineering has emerged as a pivotal tool in modern plant breeding, facilitating the transfer of desirable traits through the incorporation of alien chromosome fragments into plants. Here, we provide a comprehensive overview of the past achievements, current methodologies and future prospects of plant chromosome engineering. We begin by examining the successful integration of specific examples such as the incorporation of rye chromosome segments (e.g. the 1BL/1RS translocation), *Dasypyrum villosum* segments (e.g. the 6VS segment for powdery mildew resistance), *Thinopyrum intermedium* segments (e.g. rust resistance genes) and *Thinopyrum elongatum* segments (e.g. Fusarium head blight resistance genes). In addition to trait transfer, advancements in plant centromere engineering have opened new possibilities for chromosomal manipulation. This includes the development of plant minichromosomes via centromere‐mediated techniques, the generation of haploids through *CENH3* gene editing, and the induction of aneuploidy using KaryoCreate. The advent of CRISPR/Cas technology has further revolutionized chromosome engineering, enabling large‐scale chromosomal rearrangements, such as inversions and translocations, as well as enabling targeted insertion of large DNA fragments and increasing genetic recombination frequency. These advancements have significantly expanded the toolkit for genetic improvement in plants, opening new horizons for the future of plant breeding.

## Introduction

In a rapidly changing world, the demands on agriculture to support the growing global population are unprecedented. To maintain our current living standards, a significant leap in agricultural productivity is imperative for humanity's future (Gerland *et al*., 2014). One of the most promising avenues to achieve this is through advancements in plant chromosome engineering. The journey of plant chromosome engineering began decades ago, laying the foundation for today's advanced techniques. Initial efforts focused on incorporating alien chromosome fragments into crops, a process that has evolved dramatically over the century (Mc and Sears, 1946). Modern approaches now include precise manipulation of chromosome structures, the creation of minichromosomes and the alteration of inheritance mechanisms through technologies such as CRISPR/Cas (Puchta and Houben, 2024). These innovations have accelerated the pace of development and expanded the potential applications of chromosome engineering in agriculture. This review explores the historical and contemporary efforts in plant chromosome engineering, focusing on specific examples such as the incorporation of rye chromosome segments (e.g. the 1BL/1RS translocation), *Dasypyrum villosum* segments (e.g. the 6VS segment for powdery mildew resistance), *Thinopyrum intermedium* segments (e.g. rust resistance genes) and *Thinopyrum elongatum* segments (e.g. the 7E segment for Fusarium head blight resistance). Additionally, the review delves into the engineering of plant centromeres, highlighting the creation of plant minichromosomes through centromere‐mediated techniques, the use of *CENH3* gene editing to produce haploids, and the induction of aneuploidy using KaryoCreate (karyotype CRISPR‐engineered aneuploidy technology). The revolutionary CRISPR/Cas system's role in plant chromosome engineering is examined, particularly its applications in generating large‐scale chromosomal rearrangements, such as inversions and translocations or enabling targeted insertion of large DNA fragments and thereby increasing genetic recombination frequency. As we enter this new era of agricultural advancement, the potential applications of plant chromosome engineering appear limitless. This technology promises to revolutionize crop yields, enhance disease resistance, improve drought tolerance and address many other challenges facing global agriculture.

## Integrating alien chromosome segments into wheat to introduce new traits

The genetic basis of widely used wheat varieties in production is narrow, and the available genetic resources for breeding are relatively limited, resulting in slow progress in wheat breeding efforts. Therefore, expanding the genetic diversity of wheat varieties has become increasingly important to plant breeders (Gupta *et al*., 2022; Tyrka *et al*., 2021), who are turning to advanced techniques such as plant chromosome engineering to introduce genes from different species, genera and even more distantly related taxa into common wheat (Garland and Curry, 2022). This approach has yielded significant achievements with important practical value, including the development of wheat varieties with enhanced disease resistance, drought tolerance and yield potential.

### The application of rye in wheat genetic improvement

Rye (*Secale cereale* L., RR, 2*n* = 2*x* = 14) is a tertiary gene source for wheat and one of the first related species used in wheat genetic improvement and breeding. It possesses many excellent traits, such as drought and salt tolerance (Howell *et al*., 2014), cold resistance (Jung and Seo, 2019), rust resistance (Wehling *et al*., 2003), powdery mildew resistance (Ren *et al*., 2022; Zhu *et al*., 2023) and aphid resistance (Anderson *et al*., 2003).

In 1873, Wilson made the first attempt to hybridize wheat and rye. However, this early attempt was unsuccessful due to inadequate methods (Wilson, 1873). Despite these setbacks, persistent research and breeding efforts over the following years eventually led to the successful creation of a wheat‐rye hybrid. This milestone was followed by a significant achievement in 1888 when Rimpau synthesized the first stable amphidiploid triticale (Crespo‐Herrera *et al*., 2017; Martis *et al*., 2013).

Triticale is classified based on its chromosome ploidy levels into tetraploid (AARR, 2*n* = 4*x* = 28), hexaploid (AABBRR, 2*n* = 6*x* = 42), octoploid (AABBDDRR, 2*n* = 8*x* = 56) and decaploid (AABBDDRRR, 2*n* = 10*x* = 70) triticale (Dai *et al*., 2022). The hexaploid and octoploid varieties are particularly significant for both agricultural production and research. Hexaploid triticale is produced by hybridizing tetraploid wheat (AABB, 2*n* = 4*x* = 28) with diploid rye (RR, 2*n* = 2*x* = 14), followed by chromosome doubling to create a new allopolyploid species (Ulaszewski and Kwiatek, 2020). This variety combines all the chromosomes from the A and B genomes of wheat with the R genome of rye. Morphologically, hexaploid triticale plants are taller than common wheat and exhibit traits that are intermediate between wheat and rye. It retains rye's advantageous characteristics such as cold tolerance, drought resistance, pest and disease resistance, and high protein content, while also incorporating wheat's high yield and quality traits. Consequently, hexaploid triticale is widely used in agriculture for both grain and forage production (Li *et al*., 2015). Octoploid triticale is developed by crossing common wheat (AABBDD, 2*n* = 6*x* = 42) with rye and then doubling the chromosomes of the F_1_ hybrid (Wilson, 1873). Despite its potential, octoploid triticale has been found to be less genetically stable compared to its hexaploid counterpart (Ryan *et al*., 2018). Enhancing the genetic stability of octoploid triticale through advanced cytological techniques is a key area of ongoing research and development.

**Figure 1:**
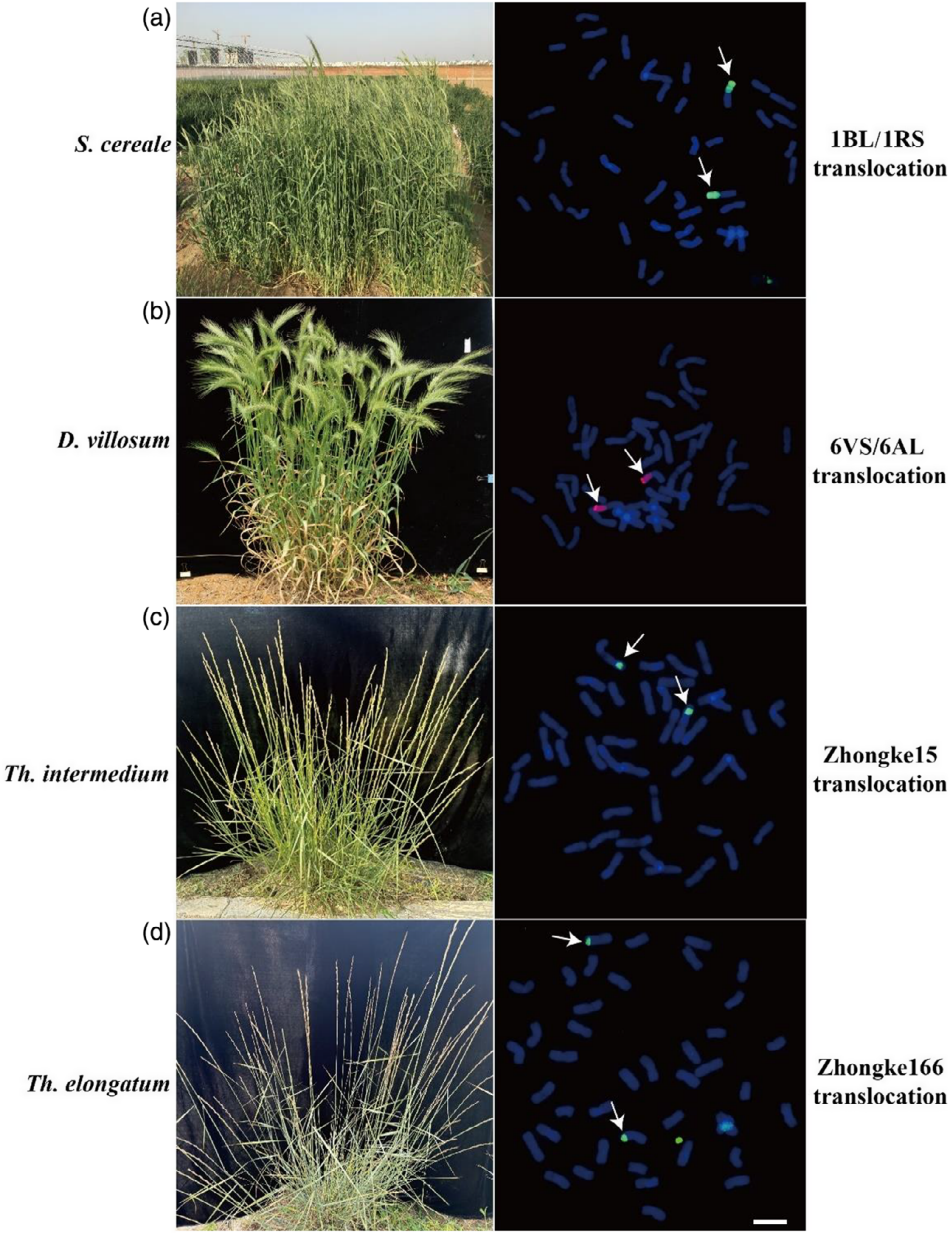
Integration of alien chromosome segments into wheat. The phenotypes of *S. cereale* (a), *D. villosum* (b), *Th. intermedium* (c) and *Th. elongatum* (d) have been instrumental in wheat genetic improvement. This includes the development of key translocations such as 1BL/1RS from *S. cereale* (a), 6VS/6AL from *D. villosum* (b), and the creation of wheat varieties like Zhongke15 (c) and Zhongke166 (d) derived, respectively, from *Th. intermedium* and *Th. elongatum*. The genomic DNA of *S. cereale* (green), *D. villosum* (red), *Th. intermedium* (green) and *Th. elongatum* (green) was used as a probe.

In the 1930s, breeders introduced the 1RS chromosome arm of rye, Petkus, into the common wheat background, replacing the 1BS chromosome arm of common wheat. This rye fragment existed in the form of T1BL/1RS. The materials created from this have been widely used in subsequent research. Among these, the most commonly used and successful application is the 1BL/1RS translocation line, followed by the 1R/1B substitution line and the 1R addition line (Crespo‐Herrera *et al*., 2017). The 1BL/1RS translocation is prevalent in wheat genomes globally and is valued for its incorporation of beneficial traits from rye into wheat (Figure 1a) (Liu *et al*., 2022; Trubacheva *et al*., 2011). The 1RS segment carries a range of beneficial genes, including resistance genes against major wheat pathogens such as leaf rust, stem rust, stripe rust and powdery mildew (Crespo‐Herrera *et al*., 2017). Additionally, it harbours genes conferring tolerance to environmental stresses like drought and salinity, along with several yield‐enhancing factors (Ren *et al*., 2017). However, there are concerns regarding the impact of this translocation on wheat quality, as it introduces specific rye‐derived proteins that can negatively affect bread‐making properties (Yan *et al*., 2005). Furthermore, the extensive use of 1BL/1RS translocation lines originating from a limited genetic pool has led to reduced genetic diversity within this segment, making wheat vulnerable to evolving pathogen populations. In recent years, changes in the pathogenicity of wheat pathogens have rendered some resistance genes ineffective, posing challenges to wheat breeding efforts (Gultyaeva *et al*., 2021; Kushnirenko *et al*., 2021; Plotnikova *et al*., 2022). To address these issues, there is a growing need to create novel 1BL/1RS translocation lines with increased genetic diversity. Furthermore, recent studies have investigated the centromere structure and function in wheat‐rye 1BL/1RS translocations. The findings revealed that the 1BL/1RS chromosome contains a hybrid centromere (Karimi‐Ashtiyani *et al*., 2021; Liu *et al*., 2024; Wang *et al*., 2017). Using CENH3 as a marker for centromere activity, it was observed that only the rye‐derived centromere portion incorporates CENH3 of wheat in the 1BL/1RS hybrid centromere (Karimi‐Ashtiyani *et al*., 2021). This research sheds light on the complex interaction between wheat and rye chromosomes in the context of translocation events. Understanding the behaviour of centromeres in such hybrid chromosomes is crucial for unravelling the mechanisms underlying chromosome stability and inheritance in wheat‐rye hybrids.

### The application of *D. villosum* in wheat genetic improvement

The genus *Dasypyrum* is primarily distributed along the Mediterranean coast and the Transcaucasus region of the Near East (Grądzielewska, 2006). It includes species such as the diploid annual *D. villosum* (VV, 2*n* = 2*x* = 14), as well as both the diploid (V^b^V^b^, 2*n* = 2*x* = 14) and tetraploid perennial *D. breviaristatum* (VVV^b^V^b^, 2*n* = 4*x* = 28) (Baum *et al*., 2014). Research on the annual *D. villosum* is significantly more extensive than on the perennial species, primarily because the annual species can hybridize with wheat, whereas the perennials have greater difficulty in hybridizing with wheat. Among the species within the genus *Triticum*, *D. villosum* can more easily hybridize with tetraploid species, such as emmer wheat, *T. dicoccum* and *T. timopheevii*. Zhukowsky obtained an amphidiploid from the cross between *T. dicoccum* and *D. villosum* (Sears, 1948). Later, McFadden and Sears (1947) also produced an amphidiploid from *T. dicoccum* and *D. villosum* using colchicine treatment (McFadden and Sears, 1947). These hybridization efforts highlighted the genetic potential of *D. villosum* for improving wheat.

*Dasypyrum villosum* not only exhibits strong resistance to various diseases such as powdery mildew and rust but also shows high resistance to most physiological races of powdery mildew. Through distant hybridization and chromosome engineering, beneficial genes from *D. villosum* have been transferred to the wheat background via chromosome translocations. Since the 1970s, researchers such as Dajun Liu and Peidu Chen have been identifying resistance genes in *D. villosum*, developing a series of wheat‐*D. villosum* introgression and substitution lines with high resistance to powdery mildew. They mapped the broad‐spectrum resistance gene *Pm21* to chromosome 6 V, and subsequently developed the wheat‐*D. villosum* translocation line T6VS/6AL through hybridization and radiation techniques (Cao *et al*., 2011; Chen *et al*., 1995; Zhang *et al*., 2023). This line has become an important germplasm resource for breeding wheat varieties resistant to powdery mildew (Figure 1b).

### The application of *Elytrigia* in wheat genetic improvement

*Elytrigia* is a perennial species that is cross‐pollinated, characterized by high protein content, stress resistance, long spikes with many florets, and close genetic relationship with common wheat, resulting in high hybridization compatibility. The genus *Elytrigia* includes five species: *E. intermedia* (*Thinopyrum intermedium*), *E. elongata* (*Thinopyrum elongatum*), *E. smithii*, *E. repens* and *E. trichophora* (Tong *et al*., 2022). Among them, the wild *Th. intermedium* and *Th. elongatum* are widely used resources.

#### *Th. intermedium*'s contribution to wheat genetic development

*Thinopyrum intermedium* (E_1_E_1_E_2_E_2_StSt or JJJ^S^J^S^StSt, 2*n* = 6*x* = 42) is a hexaploid allopolyploid species (Mahelka *et al*., 2011). It is immune to diseases such as powdery mildew, stripe rust, leaf rust and stem rust. Additionally, it exhibits notable resistance to lodging, drought, cold and saline‐alkaline conditions (Chen *et al*., 1998, 2001, 2003). The A and D genomes of wheat have high homology with the J^S^ and St genomes of *Th. intermedium*, facilitating hybridization with wheat. Therefore, using chromosome‐engineering techniques to introduce *Th. intermedium* chromosomes into the genetic background of common wheat is an effective way to improve wheat's genetic composition and broaden its genetic base (Figure 1c).

In the 1950s, Shancheng Sun used common wheat as the female parent and *Th. intermedium* as the male parent to create the Zhong series (Zhong 1 to Zhong 5) of octoploid *Trititrigia* through sexual hybridization, backcrossing and progeny selection. These hybrids displayed high resistance to stripe rust, leaf rust and stem rust and were extensively used as parents in subsequent breeding programs to produce new materials containing a large amount of wheatgrass germplasm. Liu *et al*. (2005) and Bao *et al*. (2009) developed the TE series of octoploid *Trititrigia*, including Shannong TE253 and TE257, which showed resistance to stripe rust and powdery mildew (Bao *et al*., 2009; Liu *et al*., 2005). As intermediate types between wheat and *Th. intermedium*, these octoploid *Trititrigia* can further hybridize with common wheat. Through several generations of self‐crossing, it is possible to obtain addition lines, substitution lines, translocation lines and introgression lines.

#### *Th. elongatum*'s contribution to wheat genetic development

*Thinopyrum elongatum* is a perennial herbaceous plant characterized by vigorous growth and high productivity of flowers and seeds, with strong adaptability to various environments. *Th. elongatum* can be categorized into three types: diploid (E^e^E^e^, 2*n* = 2*x* = 14), tetraploid (E^e^E^e^E^b^E^b^, 2*n* = 4*x* = 28) and decaploid (E^e^E^e^E^b^E^b^E^x^E^x^StStStSt, 2*n* = 10*x* = 70) (Heneen and Runemark, 1972; Shi *et al*., 2023). The E^e^ genome is the fundamental chromosome group of the genus *Elytrigia* and has a close genetic relationship with the wheat genome, making it conducive to distant hybridization with wheat (Dai *et al*., 2017). Research indicates that *Th. elongatum* possesses numerous excellent disease resistance genes, providing high resistance to diseases such as stripe rust, leaf rust and Fusarium head blight (FHB) (Friebe *et al*., 1993; Fu *et al*., 2012; Guo *et al*., 2023a,b; Li *et al*., 2022). It also exhibits traits such as salt tolerance and drought resistance, making it an excellent genetic resource for improving wheat traits (Li *et al*., 2019b; Wang *et al*., 2008).

In 1956, Zhensheng Li successfully developed several octoploid *Trititrigia* lines carrying excellent traits through distant hybridization between common wheat and decaploid *Th. elongatum*. Among these lines, Xiaoyan 7631 and Xiaoyan 693 are resistant to wheat streak mosaic virus and carry the *Lr24* gene (Li *et al*., 2003); Xiaoyan 7430 and Xiaoyan 68 show high resistance to wheat stripe rust; and Xiaoyan 784 is highly resistant to barley yellow dwarf virus and wheat stripe rust (Zheng *et al*., 2014).

In 1993, Fangpu Han developed hexaploid *Trititrigia* 8801, 8802 and 8803 by hybridizing durum wheat and tetraploid *Th. elongatum*. These lines exhibited high resistance to rust diseases, along with high fertility rates, plump grains with high protein content, and resistance to powdery mildew and stem rust. Additionally, 70% of the 50 derivative lines created using 8801 showed resistance to stripe rust (Mo *et al*., 2017). Fangpu Han also found that hexaploid *Trititrigia* had very good resistance to FHB. Diploid *Th. elongatum* also shows high resistance to FHB, and recent studies indicate that tetraploid *Th. elongatum* evolved from diploid *Th. elongatum* through homologous doubling (Shi *et al*., 2023). Through pollen irradiation of the diploid *Th. elongatum* addition line (CS‐7EL) and continuous multi‐year and multi‐location field and greenhouse FHB inoculation, 843 stable translocation lines were identified. These were further refined through backcrossing and selection, resulting in several new wheat lines that combine FHB resistance with excellent agronomic traits. Among them, Zhongke 166 (a 7D chromosome segment translocation line) and Zhongke 1878 (a 6D chromosome segment translocation line) have been certified by governmental regulators in China and are now considered important new varieties resistant to FHB (Figure 1d) (Guo *et al*., 2023a,b).

## Methods for developing wheat‐alien species translocation lines

**Figure 2:**
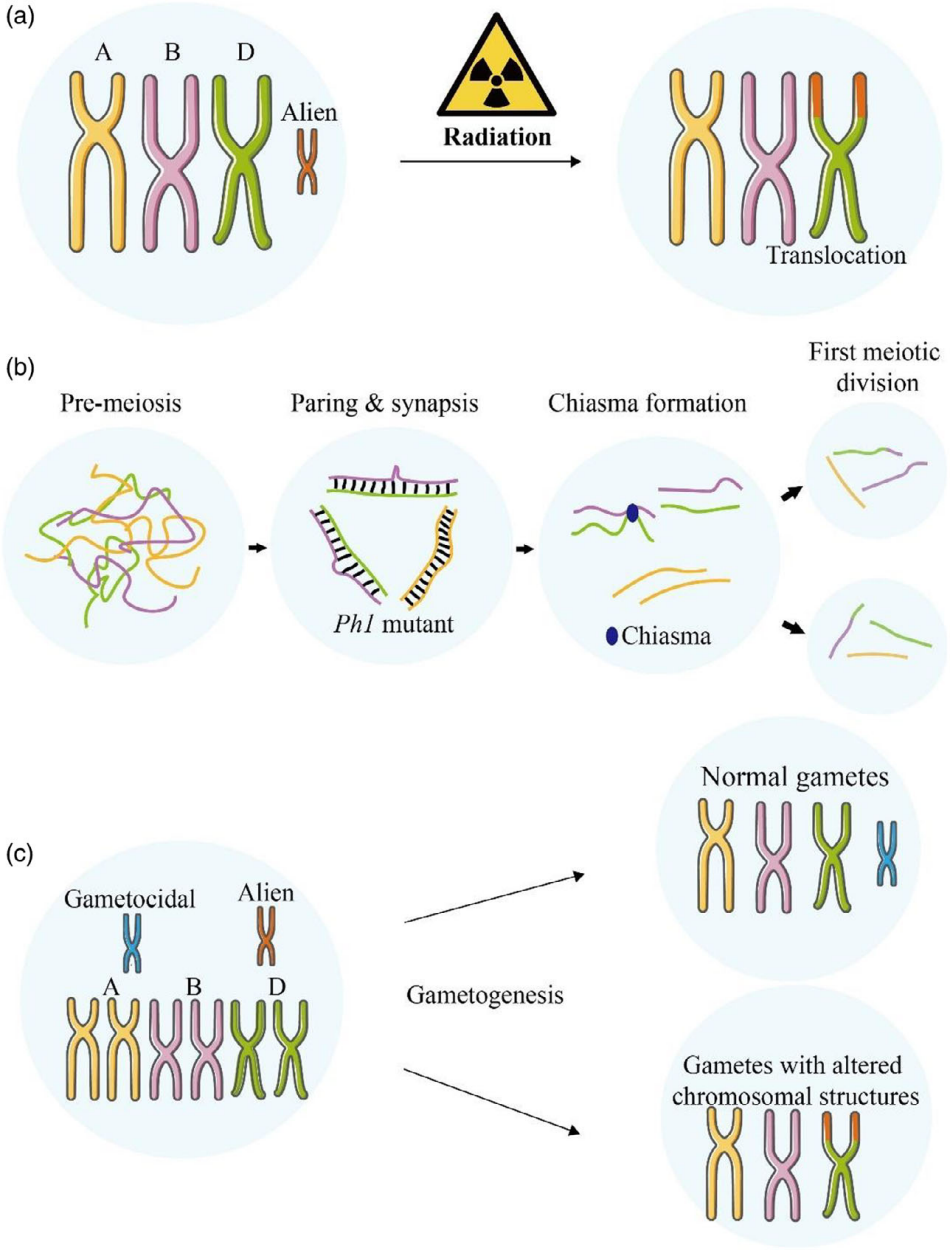
Methods for developing wheat‐related species translocation lines. (a) Ionizing radiation: This method induces chromosome breaks and facilitates the exchange of segments between wheat and related species. (b) A schematic representation of chromosome pairing regulated by *Ph1*. (c) Gametocidal chromosomes from related species induce chromosome breakage in wheat, leading to the preferential retention and incorporation of alien chromosome segments during gametogenesis.

Alien chromosomal lines from wheat‐related species, including amphidiploids, alien addition lines and alien substitution lines, carry not only many beneficial genes from related species but also many redundant or deleterious genes linked to the beneficial ones. This makes it difficult to widely promote their use in wheat production. Therefore, creating and breeding small‐segment translocation lines carrying target beneficial genes from related species, especially minimal segment introgression lines, is an important and effective method for utilizing the beneficial genes from related species. Currently, the methods for developing wheat‐alien chromosome translocation lines include ionizing radiation, the *Ph1* (pairing homologous 1) gene and gametocidal chromosomes (Figure 2).

### Ionizing radiation

The use of ionizing radiation to induce translocation lines is a powerful technique in plant breeding and genetic research. Depending on the radiation field, ionizing radiation can be classified into several types: α radiation, β radiation, γ radiation and neutron radiation (Hendee, 2010). Wheat can be treated with ionizing radiation at various stages, including seeds, young spikes during meiosis and mature pollen. When plants are treated with ionizing radiation, their chromosomes experience random breaks. These breaks can result in various structural changes as the chromosomes rejoin in new configurations. Such structural variations include deletions, inversions and translocations (Figure 2a).

In 1956, Sears first used X‐ray treatment on wheat‐*Aegilops umbellulata* monosomic addition lines and obtained a translocation line, T6BS·6BL‐6UL, carrying a segment of the *A. umbellulata* chromosome. This successfully introduced the leaf rust resistance gene *Lr9* from *A. umbellulata* into the wheat background (Sears, 1956). Zhensheng Li and colleagues utilized wheat‐*Th. elongatum* hybrids, followed by backcrossing, and subsequently applied laser radiation to the wheat‐*Th. elongatum* hybrid progeny. This process resulted in the development of the backbone wheat parent variety, Xiaoyan 6. Xiaoyan 6 is a wheat‐*Th. elongatum* translocation line that has been widely cultivated, setting a precedent for the large‐scale adoption of distant hybridization in wheat breeding. Fangpu Han used ^60^Co‐γ rays to treat wheat‐*Th. elongatum* 7EL telosome addition lines and obtained wheat‐*Th. elongatum* translocation lines with high resistance to FHB, which have played a significant role in wheat breeding (Fu *et al*., 2012; Guo *et al*., 2023a,b).

### *Ph1* induction

The *Ph1* in common wheat is located on the long arm of chromosome 5B. Its presence promotes pairing between homologous chromosomes while inhibiting pairing between homologous chromosomes, thereby maintaining the genetic stability and fertility of the wheat genome (Riley and Chapman, 1958; Zhang *et al*., 2020). If the *Ph1* gene is deleted or suppressed, the recombination frequency between homologous chromosomes significantly increases, which can induce the production of wheat‐alien species translocation lines or introgression lines (Figure 2b).

Previously, various types of translocation lines have been created using the *Ph1* gene. For instance, Sears *et al*. crossed *Agropyron elongatum* substitution lines with a common wheat 5B‐nullisomic line, producing a BC_1_F_2_ population and subsequently screening two wheat‐*A. elongatum* translocation lines, namely 3D/3Ag and 7D/7Ag (Sears, 1956). Similarly, Niu *et al*. utilized the *Ph1* mutant to induce two small‐segment translocation lines carrying the stem rust resistance gene *Sr43* (Niu *et al*., 2008). Li *et al*. used the Chinese Spring *Ph1* homozygous recessive mutant to cross with a wheat‐*D. villosum* T6AS/6VL homozygous translocation line, obtaining an F_1_ population from which they screened six types of homozygous structural variants involving the 6VL chromosome arm (Li *et al*., 2019a).

Despite the significant progress in using the *Ph1* gene to induce translocation lines, this technique has limitations. First, the types of translocations are relatively few. Second, most studies utilize the Chinese Spring *Ph1* homozygous recessive mutant, which typically exhibits tall, weak stems, leading to severe lodging and poor agronomic traits. As a result, wheat‐alien species chromosomal introgression lines created using the Chinese Spring *Ph1* homozygous recessive mutant have not been widely adopted in production. These introgression lines need to be repeatedly backcrossed and self‐crossed with widely cultivated varieties to select materials with desirable agronomic traits in their progeny.

In response to these limitations, recent advances have focused on the *TaZIP4‐B2* gene (located within the *Ph1* region) as a more precise and targeted method for inducing homeologous recombination while avoiding the undesirable side effects seen with the broader *Ph1* deletion mutants (Martín *et al*., 2018). Studies have shown that *TaZIP4‐B2* is crucial in promoting homologous pairing and synapsis while also suppressing crossovers between related homeologous chromosomes. In contrast to the large *ph1b* deletion mutant (which removes 59.3 Mb and disrupts 1187 genes), *TaZIP4‐B2* loss‐of‐function mutants (e.g. the Tazip4‐B2 line) retain the essential homologous pairing function but allow increased homeologous crossovers, without compromising wheat fertility or agronomic traits (Draeger *et al*., 2023; Martín *et al*., 2021).

For instance, *Tazip4‐B2* mutants crossed with *Aegilops variabilis* and other wheat relatives exhibit increased homeologous crossover, facilitating the creation of translocation lines more efficiently than the traditional *Ph1* mutants (Rey *et al*., 2018). This opens the door to broader applications in wheat breeding, particularly for incorporating useful genetic material from wild species into cultivated wheat varieties. Thus, utilizing *TaZIP4‐B2* in future breeding programs could offer a more effective alternative for generating stable translocation lines while improving the introgression of desirable traits into modern wheat cultivars.

### Gametocidal chromosomes

Gametocidal chromosomes are a type of chromosome with a preferential transmission effect (Figure 2c). They allow gametes containing these chromosomes to be normally fertile while inducing breakage and reattachment in other chromosomes in gametes lacking these chromosomes, resulting in structural variations such as deletions and translocations (Miller *et al*., 1982; Said *et al*., 2024). Therefore, gametocidal chromosomes can be used to create wheat‐alien species translocation lines, offering an effective, easy‐to‐operate and safer mutagenesis method compared to chemical mutagens (Endo, 2007).

In 1957, Cameron and Moav first discovered gametocidal chromosomes in tobacco. Subsequent discoveries followed in maize and tomato (Maguire, 1963; Rick, 1966). Endo and Tsunewaki found gametocidal chromosomes in *Aegilops* in 1975 (Endo, 1985). In a wheat genetic background, wheat lines with only one gametocidal chromosome can produce two types of gametes: those with and those without the gametocidal chromosome. Gametocidal chromosomes kill gametes without them by causing chromosomal abnormalities, including fragments, dicentric chromosomes and chromosome bridges (Said *et al*., 2024).

There are many cases where gametocidal chromosomes have introduced desirable genes from wheat‐related species into common wheat, resulting in various types of wheat‐alien species translocation lines. For example, Masoudi *et al*. successfully translocated 1R chromosome segments from rye into wheat chromosomes 2A, 2D, 3D, 5D and 7D using the gamete‐killing 3C system (Masoudi‐Nejad *et al*., 2002). Sakata *et al*. used this method to obtain progeny materials of a wheat‐barley 4H addition line with the gamete‐killing chromosome 2C and found that the rearranged barley 4H chromosomes exhibited complex structural variations (Sakata *et al*., 2010). Tsuchida *et al*. crossed a Chinese Spring‐2C disomic addition line with a Chinese Spring‐rye disomic addition line, obtained F_1_ hybrids, backcrossed the F_1_ hybrids with the parents to obtain F_2_ progeny with 45 chromosomes and found numerous plants carrying rearranged 1Ri chromosomes (Tsuchida *et al*., 2008). Overall, gametocidal chromosomes have successfully induced translocations and deletions in various systems, including wheat‐*Thinopyrum*, Chinese Spring‐barley disomic addition lines, Chinese Spring‐rye disomic addition lines and *Agropyron*.

## Modern advances in plant chromosome engineering techniques

Plant chromosome engineering has historically relied on methods such as ionizing radiation, the *Ph1* gene, and gametocidal chromosomes to develop wheat‐alien chromosome translocation lines. While these techniques were groundbreaking at their inception and remain in use today, they have limitations in terms of precision and efficiency. However, with the deepened understanding of centromere functionality and the optimization of CRISPR/Cas9 technology, plant chromosome engineering has made significant strides. The current advancements enable more precise manipulation of chromosomes, allowing for targeted modifications and improved integration of desirable traits. This progress not only enhances the efficiency of developing new crop varieties but also opens up new possibilities for genetic research and agricultural biotechnology.

### Manipulating centromeres for the generation of haploid inducers and aneuploidy

**Figure 3:**
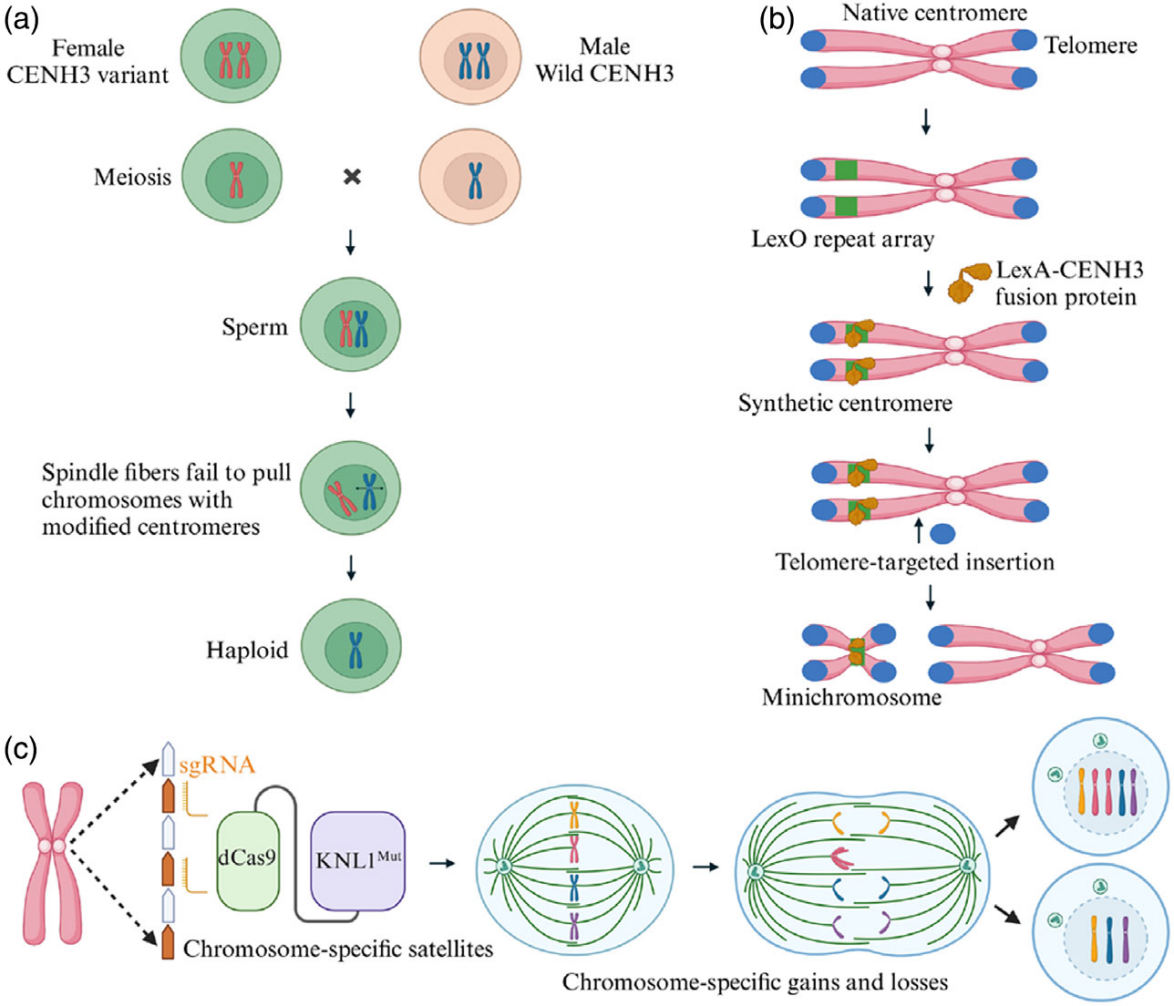
Manipulating centromeres for the generation of haploid inducers, minichromosomes and aneuploidy. (a) Haploid generation using CENH3 variants: Haploids are achieved by employing CENH3 variants that either modify or compromise centromere integrity. When these variant lines are crossed with wild‐type counterparts, the resulting offspring exhibit haploid formation due to the elimination of chromosomes from one parent. (b) Top‐Down strategy for synthetic chromosome generation using the LexO‐LexA system. A LexA‐CENH3 fusion protein is tethered to a chromosomal LexO repeat array, initiating the formation of a *de novo* centromere. By combining *de novo* centromere formation with targeted telomere seeding near the tandem repeats, a minichromosome can be released. (c) Schematic representation of the KaryoCreate system for generating chromosome‐specific aneuploidies. The system utilizes co‐expression of sgRNA targeting chromosome‐specific CENPA‐binding α‐satellite repeats and a dead Cas9 (dCas9) fused to a mutant KNL1. Upon introduction into cells, the system facilitates controlled mis‐segregation of the targeted chromosomes, resulting in precise gains or losses of the specific chromosome in the cellular progeny.

CENH3 is a crucial histone H3 variant found exclusively in the functional centromeres of eukaryotes. It plays a pivotal role in the recruitment and stabilization of centromere protein complexes, which are essential for chromosome segregation during cell division and the positioning of centromeres on chromosomes (Liu *et al*., 2021, 2023). Structurally, CENH3 consists of an N‐terminal tail, which is a target for post‐translational modifications, and a C‐terminal histone fold domain (HFD) (Cui *et al*., 2024). Importantly, point mutations or sequence replacements in CENH3 can compensate for cenh3 mutations, leading to the formation of haploid inducer lines that produce haploid offspring when crossed with wild‐type plants (Figure 3a) (Marimuthu *et al*., 2021).

In 2010, Ravi and Chan achieved a breakthrough by modifying CENH3 in Arabidopsis. They created two constructs: one with green fluorescent protein fused to the N‐terminus of CENH3 (GFP‐CENH3), and another where the N‐terminus of CENH3 was replaced by that of histone H3.3, also fused with GFP (GFP‐tailswap). Both constructs successfully restored the wild‐type phenotype in Arabidopsis cenh3‐1 mutants and induced haploid plants when crossed with different ecotypes (Ravi and Chan, 2010). This discovery catalysed extensive research into CENH3‐mediated haploid induction across various crops. Building on this foundation, in 2015, Anne Britt's team discovered a point mutation causing a single amino acid change in the HFD of CENH3 could reduce its centromere‐binding function. Crossing this mutant with wild‐type Arabidopsis produced haploid offspring (Kuppu *et al*., 2015). They subsequently identified 31 additional CENH3 alleles with single amino acid substitutions from EMS‐mutagenized Arabidopsis progeny (Kuppu *et al*., 2020) offering crop breeders with more options for developing haploid induction lines. In maize, haploids were generated by crossing cenh3 null mutant heterozygotes (Kelliher *et al*., 2016; Wang *et al*., 2021). In wheat, gene editing of *TaCENH3α* created a mutant with a restored frameshift heterozygous state for *TaCENH3α‐A* and knockouts of *TaCENH3α‐B* and *TaCENH3α‐D*, achieving a haploid induction rate of up to 8% (Lv *et al*., 2020). Esteban Bortiri's team further developed a CENH3 haploid induction system with a dominant colour marker gene, facilitating the rapid conversion of maize inbred lines into males‐sterile lines (Bortiri *et al*., 2024). In *Brassica* crops, Han *et al*. used CRISPR‐Cas9 to edit *BoCENH3*, creating a paternal haploid inducer line that mediated cytoplasmic replacement, achieving a breakthrough in the ‘one‐step’ creation of cytoplasmic male sterility lines (Han *et al*., 2024). Additionally, RNAi‐mediated downregulation of *AcCENH3* induced haploid production in onions (Manape *et al*., 2024).

Beyond CENH3, mutations in other centromere function‐related genes, such as *VIM1* (Marimuthu *et al*., 2021) and *KNL2* (Ahmadli *et al*., 2023), have been shown to increase haploid induction rates. The integration of genome editing technologies like CRISPR/Cas9 with CENH3‐mediated haploid induction has significantly enhanced the precision and efficiency of creating superior haploid induction lines. This approach allows for the rapid development of homozygous inbred lines with diverse genetic backgrounds within two generations, greatly advancing crop‐breeding technologies and driving progress in biological breeding.

In recent advancements, a novel system called KaryoCreate has been developed, enabling precise generation of chromosome‐specific aneuploidies. This system functions by co‐expressing a single‐guide RNA (sgRNA) targeting chromosome‐specific CENPA (CENH3 in plants)‐binding α‐satellite repeats alongside a dCas9 fused to a mutant KNL1. The authors designed unique and highly specific sgRNAs for 19 of the 24 human chromosomes, allowing for controlled mis‐segregation and subsequent gains or losses of the targeted chromosome in cellular progeny. Notably, the system achieved an average efficiency of 8% for chromosomal gains and 12% for losses (up to 20%), which was validated across 10 different chromosomes (Bosco *et al*., 2023). The potential application of KaryoCreate in plants offers exciting possibilities for advancing chromosome engineering and plant breeding. In plants, aneuploidy can have significant effects on phenotypic traits such as growth, development and stress resistance. Adapting KaryoCreate for plant systems would involve targeting plant‐specific centromeric repeats, using CENH3 to induce controlled mis‐segregation events. This approach could allow for the precise manipulation of individual chromosomes, enabling researchers to study gene dosage effects, chromosomal stability and their impact on important agronomic traits.

The potential application of KaryoCreate in plants offers exciting possibilities for advancing chromosome engineering and plant breeding. To create translocation lines, it is essential to first obtain monosomic alien addition lines (MAALs). Due to the genomic instability of these alien addition lines, they are often difficult to utilize directly in breeding. Therefore, they serve as intermediate bridge materials for constructing a series of translocation lines through methods such as homologous recombination, radiation or gametocidal agents. This process typically involves interspecific or intergeneric hybridization to obtain F_1_ hybrids, followed by colchicine treatment to double the F_1_ to produce double haploids. The double haploids are then backcrossed with one parent over several generations to obtain the monosomic addition lines. This method is time‐consuming and labour‐intensive, and it does not guarantee the creation of MAALs that contain a complete set of alien chromosomes.

In this context, KaryoCreate technology can significantly enhance efficiency. For instance, specific chromosomes in the double haploids can be targeted using KaryoCreate technology to induce their loss, thereby retaining certain chromosomes and achieving the rapid creation of MAALs. This process requires a detailed understanding of the centromere structure of the alien chromosomes to ensure specificity when designing sgRNAs.

### Plant minichromosomes

Plant minichromosomes are small‐sized chromosomes created by deleting parts of natural chromosomes (Murata, 2014). They are designed to carry additional genetic material without disrupting the existing structure and function of the host genome. Typically ranging from a few hundred kilobases to a few megabases, these minichromosomes include essential elements such as centromeres, telomeres and origins of replication to ensure the stable maintenance and correct segregation during cell division (Murata, 2014). Plant minichromosomes hold significant potential for trait stacking and genetic improvement. Unlike traditional genetic modification methods, which integrate alien genes into the host genome and may lead to unpredictable effects, minichromosomes can serve as vectors to carry and stably express a large number of alien genes (Kan *et al*., 2022). This enables targeted modification and precise control of biological systems, enhancing disease and pest resistance, increasing nutritional content and improving environmental tolerance.

There are several methods for creating plant minichromosomes: (1) Early techniques involved using physical radiation to generate minichromosomes. For instance, X‐ray or gamma‐ray irradiation of maize pollen, as well as translocations involving B‐9 and duplication 9S, were employed to produce maize minichromosomes (Kaszás and Birchler, 1998). However, these minichromosomes often suffered from issues such as unstable transmission during meiosis or mitosis, inability to carry site‐specific recombination sites and limited effectiveness in expressing heterologous sequences, which constrained their application. (2) Telomere‐mediated truncation is another method. This approach involves integrating cloned telomeric repeats to remove chromosome arms and native telomeric sequences, replacing them with transgenes and new telomeric sequences to create modifiable minichromosomes. For example, Arabidopsis telomere sequences cloned using pAtT4 have been utilized for this purpose (Richards and Ausubel, 1988). Birchler's team pioneered this technique in maize by using Arabidopsis‐type telomere repeats to shorten maize chromosomes (Swyers *et al*., 2016; Yu *et al*., 2006). Constructs such as pWY76 and pWY86, which include Cre/lox or FLP/FRT site‐specific recombination cassettes, were later employed for chromosomal truncation (Yu *et al*., 2007). Birchler's group also used telomere truncation and Cre recombinase to generate maize minichromosome carrying transgenes flanked by loxP sites, creating customizable chromosome platforms (Gaeta *et al*., 2013). This method has been successfully applied in various species, including Arabidopsis (Nelson *et al*., 2011; Teo *et al*., 2011), rice (Xu *et al*., 2012), barley (Kapusi *et al*., 2012), Brassica napus (Yan *et al*., 2017; Yin *et al*., 2021) and wheat (Yuan *et al*., 2017). Although truncated chromosomes are transmissible through sexual reproduction, their inheritance often deviates from Mendelian expectations. (3) Manipulating naturally minichromosomes‐supernumerary B chromosomes. B chromosomes, present in many plants, including maize (Randolph, 1941), rye (Boudichevskaia *et al*., 2022) and wheat (Ruban *et al*., 2020), generally have minimal impact on plant growth and development, making them ideal candidates for modification. By incorporating desired genes and regulatory elements, B chromosomes can be transformed into functional minichromosomes, providing an effective platform for alien gene vectors. This transformation enables the stable incorporation and expression of numerous alien genes without disrupting the plant's normal physiological functions. B chromosomes are also excellent targets for telomere‐mediated truncation (Birchler and Swyers, 2020). In many plants, complete B chromosomes exhibit nondisjunction (Banaei‐Moghaddam *et al*., 2012; Blavet *et al*., 2021). Using telomere‐mediated truncation to remove the regions that control nondisjunction allows the modified B chromosome minichromosomes to segregate normally (Birchler *et al*., 2016). (4) The LexA‐LexO binding system (Figure 3b). Kelly Dawe and colleagues used this system to target CENH3 to synthetic repeat sequences, activating mitosis and forming stable centromeres in maize. They observed chromosome fragments in late‐stage nuclei, which could be transmitted to the next generation (Dawe *et al*., 2023). (5) The Cre/lox system. Minoru Murata and his team used the Cre/LoxP and Activator/Dissociation element systems to generate a 2.85 Mb artificial ring chromosome in Arabidopsis. This minichromosome originated from the centromeric edge of the long arm of chromosome 2, demonstrating the potential of the Cre/lox system in minichromosome engineering (Murata *et al*., 2013).

While minichromosomes have provided a useful approach in plant chromosome engineering, they often depend on the existing chromosomal architecture, which may impose constraints on the design and function of the synthetic elements. In contrast, *de novo* chromosome synthesis adopts a bottom‐up approach, constructing entirely new chromosomes from scratch. This method has shown significant progress in simpler organisms like yeast, where synthetic chromosomes can be designed with customized features, offering more flexibility and precision (Zhao *et al*., 2023). The question remains, how can this be applied to plants? A recent breakthrough in the moss *Physcomitrium patens* provides some answers. Researchers demonstrated the successful *de novo* assembly and integration of a synthetic chromosome fragment in moss. The team redesigned the chromosome sequence, significantly simplifying the genome by removing 55.8% of endogenous sequences and adding artificial tags. They replaced about one‐third of a chromosome arm with this synthetic sequence, and the engineered plants were able to grow normally, with the synthetic region re‐establishing its epigenetic landscape (Chen *et al*., 2024). This study represents an important step forward in *de novo* chromosome assembly in plants, highlighting its potential to overcome the limitations of top‐down approaches and paving the way for future applications in synthetic plant biology.

### CRISPR‐mediated plant chromosome engineering

The CRISPR/Cas system has emerged as a versatile tool for plant genome engineering, capable of inducing a wide range of genomic modifications beyond simple deletions and mutations. Notably, this technology has demonstrated remarkable potential in generating large‐scale chromosomal rearrangements, such as inversions and translocations, as well as enabling targeted insertion of large DNA fragments and increasing genetic recombination frequency (Beying *et al*., 2020; Rönspies *et al*., 2021; Schmidt *et al*., 2020; Schwartz *et al*., 2020; Zou *et al*., 2024). These advancements have been particularly transformative for plant genetics and breeding.

**Figure 4:**
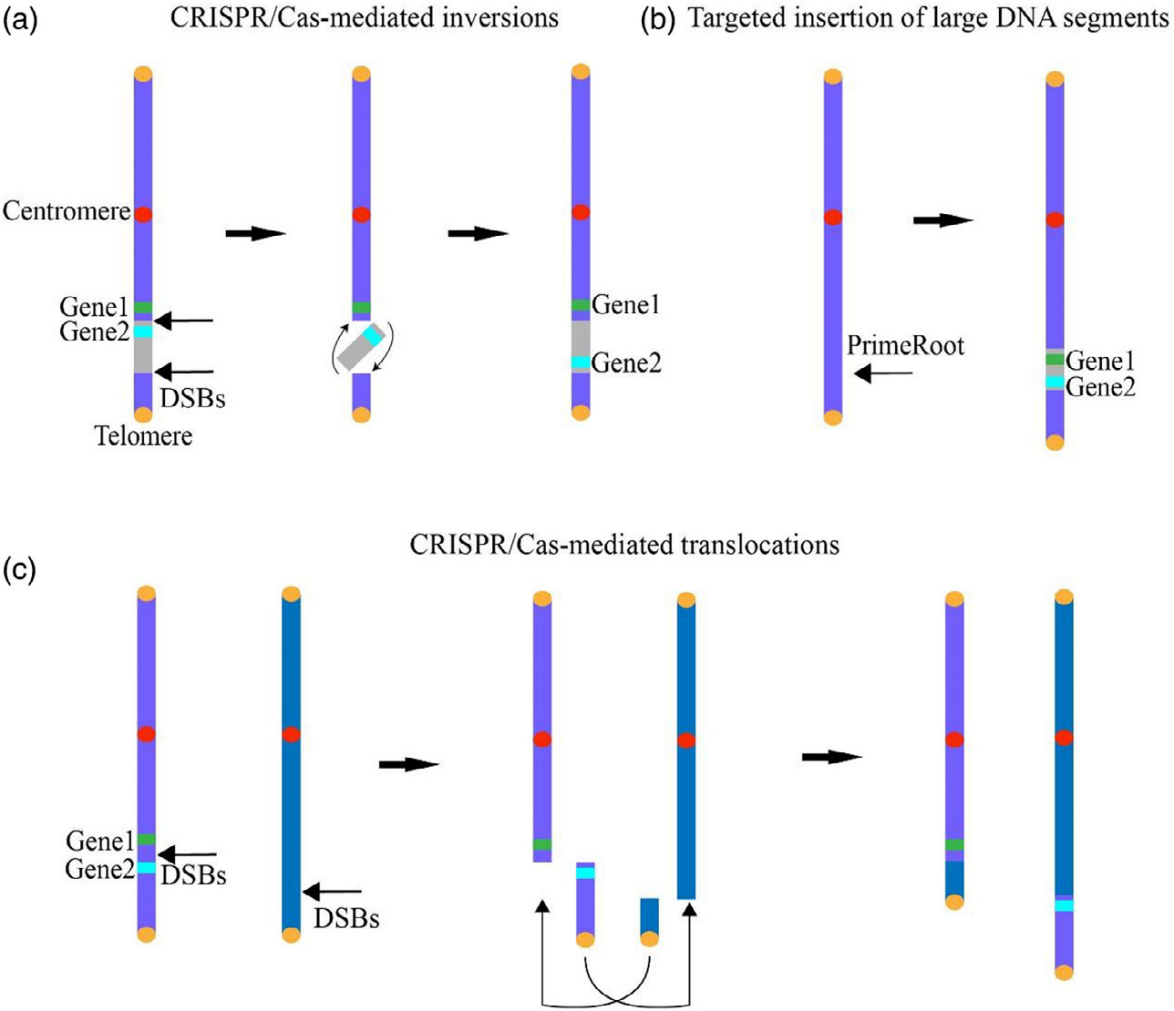
CRISPR‐mediated plant chromosome engineering. (a) Targeted chromosomal inversions can effectively disrupt genetic linkage between two closely associated genes. This process involves inducing a DSB at a specific location between the two linked genes. Following the induction of the inversion, the physical separation of these genes occurs, breaking their genetic linkage. (b) The PrimeRoot system is utilized for the precise insertion of large DNA segments, including genes of interest, into a target genome. (c) CRISPR‐Cas9‐induced DSBs effectively separate linked genes, enabling independent trait segregation and enhancing plant agronomic characteristics.

The development of CRISPR/Cas technology has revolutionized plant chromosome engineering by providing efficient nucleases like Cas9 and Cas12a, which can induce double‐strand breaks (DSBs) at almost any genomic position. This precise ability has enabled researchers to achieve various chromosomal rearrangements, such as inversions, with unprecedented efficiency and accuracy (Gao, 2021; Nasti and Voytas, 2021). For instance, initial studies with CRISPR/Cas9 demonstrated the induction of kb‐sized inversions in *Arabidopsis*, though the efficiency remained lower compared to deletions (Schmidt *et al*., 2019). This proof‐of‐concept laid the groundwork for more sophisticated chromosome engineering projects. Subsequent optimizations, such as egg‐cell‐specific expression of SaCas9 and enhanced screening protocols, significantly improved the efficiency and scale of CRISPR‐mediated inversions (Rönspies *et al*., 2022a; Schmidt *et al*., 2020). Notably, a landmark achievement was the reversion of the 1.1 Mb hkS4 inversion in *Arabidopsis*, where crossing experiments confirmed restored genetic exchange in a region previously isolated for millennia (Schmidt *et al*., 2020). Furthermore, the induction of larger inversions, such as a 17 Mb inversion on chromosome 2 of *Arabidopsis*, demonstrated a significant reduction in crossover frequency, suggesting the potential to preserve advantageous genetic linkages (Rönspies *et al*., 2022b). Conversely, the reversion of a 75.5 Mb inversion in maize facilitated genetic exchange in previously inaccessible regions, underscoring the versatility of this approach (Schwartz *et al*., 2020). Beyond modulating genetic exchange, chromosomal inversions have shown utility in regulating gene expression. The ‘knock up’ technique demonstrated in rice, where a 0.9 Mb inversion was induced to achieve a promoter swap, resulted in a massive increase in target gene expression (Lu *et al*., 2021). Such capabilities highlight the utility of chromosomal inversions in both structural and functional genomics, offering diverse applications in plant breeding (Figure 4a).

In addition to inversions, CRISPR/Cas systems have also been employed to induce chromosomal translocations, offering another powerful tool to break genetic linkages (Figure 4c). While both classical and alternative nonhomologous end joining (NHEJ) pathways efficiently repair somatic DSBs in plants (Gehrke *et al*., 2022), NHEJ‐based translocations can be induced to alter chromosome structure and gene linkages (Beying *et al*., 2020). In a proof‐of‐concept experiment in *Arabidopsis*, researchers demonstrated the reciprocal exchange of chromosome arm parts. Using a similar setup to that used for inversions, they achieved exchanges between chromosomes 1 and 2, as well as 1 and 5 (Beying *et al*., 2020). However, the frequencies of these translocations were several‐fold lower than those reported for intrachromosomal inversions. Interestingly, translocation frequencies could be enhanced by blocking the classical NHEJ pathway, a finding previously reported for inversions (Beying *et al*., 2020; Schmidt *et al*., 2019). The lower efficiency of translocations compared to inversions can be explained by the classical NHEJ pathway's role in keeping the ‘correct’ ends of a DSB in close proximity, thus avoiding misjoining of ‘wrong’ ends that could lead to genome instability. While blocking classical NHEJ might increase chromosome engineering efficiency, it is important to consider that general loss of this pathway can cause genetic instability (Beying *et al*., 2020).

In addition to these advancements, the precise manipulation of large DNA segments has emerged as a significant breakthrough (Li *et al*., 2024). While base editing and prime editing have primarily focused on base‐pair‐level changes, recent innovations have expanded CRISPR technology to accommodate larger‐scale modifications. Prime editing‐based dual pegRNA systems have enabled the insertion and deletion of large DNA segments with improved efficiency and precision. For instance, the PrimeRoot system has achieved precise insertions of up to 11.1 kb in plants such as rice and maize. This method uses dual‐ePPE to target specific genomic sites, allowing for the seamless integration of large DNA sequences without relying on endogenous DNA‐repair mechanisms, which are often limiting factors in traditional editing methods (Figure 4b) (Sun *et al*., 2024). Furthermore, transposase‐based methods have shown great promise in the precise insertion of large DNA segments. Systems like FiCAT (find and cut‐and‐transfer) utilize inactivated PiggyBac transposases coupled with Cas9 to increase targeting specificity, reducing off‐target effects and enhancing the precision of large DNA insertions (Pallarès‐Masmitjà *et al*., 2021). Similarly, site‐specific recombinase systems, such as PASTE (programmable addition via site‐specific targeting elements), have demonstrated the capability to insert large DNA segments up to 36 kb in mammalian cells and are being adapted for use in plants (Anzalone *et al*., 2022; Yarnall *et al*., 2023). These recombinase‐based systems do not rely on the cell's DNA‐repair mechanisms, making them highly efficient for large‐scale genomic modifications.

Finally, CRISPR‐mediated DSBs have been shown to promote genetic recombination by increasing crossover (CO) frequency, particularly in regions of the genome that typically experience low recombination rates (Samach *et al*., 2023). One prominent example is in tomato, where targeted DSBs induced by CRISPR/Cas9 led to a significant increase in homologous crossover events, thereby enhancing genetic diversity in these regions (Filler Hayut *et al*., 2017). In *Arabidopsis*, certain genes such as *FANCM* and *RECQ4* play crucial roles in regulating CO frequency by suppressing crossover events. Mutations in these genes have been extensively studied for their effects on crossover rates (Fernandes *et al*., 2018). In the future, CRISPR technology could be used to mutate these genes to increase CO frequency in plants. Additionally, CRISPR could be employed to alter DNA methylation patterns, a mechanism that regulates gene expression and CO distribution. Studies in *Arabidopsis* mutants have demonstrated that loss of CG methylation leads to changes in CO distribution (Melamed‐Bessudo and Levy, 2012; Mirouze *et al*., 2012), suggesting that manipulating methylation through CRISPR could be another avenue to increase recombination rates.

These technologies offer promising tools for achieving substantial genetic modifications, enabling the precise stacking of beneficial traits and improving overall plant performance under various environmental conditions. As these methods continue to be refined and optimized, they are expected to significantly enhance the precision and efficiency of plant breeding programs, ultimately leading to the development of crop varieties with superior traits and improved adaptability (Capdeville *et al*., 2023; Wang and Doudna, 2023).

## Conclusions and future prospects

Plant chromosome engineering has become an essential tool in modern plant breeding, enabling the precise transfer and integration of desirable traits from alien species into crops. The incorporation of rye chromosome segments like the 1BL/1RS translocation, and the introduction of resistance genes from species such as *D. villosum*, *Th. intermedium* and *Th. elongatum* have demonstrated the significant impact of chromosome engineering on enhancing crop resilience and productivity. Progress in centromere engineering, including the development of plant minichromosomes and the generation of haploids through CENH3 editing, has further expanded the possibilities for chromosomal manipulation, providing new avenues for plant genetic improvement. The advent of CRISPR/Cas technology revolutionized the field, offering unprecedented precision in chromosome engineering. This technology has facilitated complex genomic modifications, including chromosomal inversions, translocations and the insertion of large DNA segments, thereby massively enhancing the potential for crop improvement.

However, the challenges that remain are no less significant and must be addressed to fully realize the potential of plant chromosome engineering. First, CRISPR‐mediated chromosomal rearrangements, particularly translocations, remain inefficient. While inversions and deletions have shown moderate success, translocations occur at a low frequency, often requiring extensive screening to identify successful events. This inefficiency is largely due to the classical NHEJ repair pathway, which tends to keep the correct ends of DSBs in close proximity, preventing misjoining and reducing the likelihood of translocations. Another challenge is the risk of genetic instability associated with large‐scale chromosomal modifications. Inducing multiple DSBs or large inversions can lead to unintended genome rearrangements or off‐target effects, which compromises the stability and viability of the engineered plants. Finally, the application of advanced chromosome engineering techniques is still limited to model plants like Arabidopsis and rice, with less progress made in more complex or economically important crops.

To overcome these challenges, future research should focus on improving the efficiency and specificity of chromosome engineering tools. For translocations, this could involve developing more efficient nucleases or selectively inhibiting specific DNA‐repair pathways to increase the desired rearrangement outcomes. Additionally, more precise editing tools must be developed, along with stringent screening protocols, to reduce genetic instability and off‐target effects. Advanced techniques like base editing and prime editing could offer more refined control over genetic modifications, lowering the risk of unintended consequences. To broaden the applicability of these technologies, species‐specific CRISPR systems and optimized delivery methods should be developed to ensure efficient editing across diverse crops. By integrating chromosome engineering with traditional breeding and high‐throughput screening methods, the development of stable, high‐yielding crop varieties can be accelerated, ultimately enabling chromosome engineering to support sustainable agriculture and enhance crop resilience in the face of local and global environmental challenges.

## Conflict of interest

The authors declare no conflict of interest.

## Author contributions

All authors contributed to the review article. F.H. and Y.L. discussed the manuscript. Y.L., Q.L., C.Y. and F.H. wrote the manuscript. Y.L. and C.L. prepared the figures. All authors approved the final version of the manuscript.

## Data Availability

Data sharing not applicable to this article as no datasets were generated or analysed during the current study.
